# The Influence of Perceived Organizational Support on Police Job Burnout: A Moderated Mediation Model

**DOI:** 10.3389/fpsyg.2020.00948

**Published:** 2020-05-26

**Authors:** Xiaoqing Zeng, Xinxin Zhang, Meirong Chen, Jianping Liu, Chunmiao Wu

**Affiliations:** ^1^School of Psychology, Jiangxi Normal University, Nanchang, China; ^2^Department of Education, Nanchang Normal University, Nanchang, China

**Keywords:** perceived organizational support, job burnout, job satisfaction, regulatory emotional self-efficacy, moderated mediation

## Abstract

**Objective:**

Based on the theory of perceived organizational support (POS), conservation of resource (COR) and job demands-resources (JD-R) model, this study establishes a moderated mediation model to test the role of job satisfaction in mediating the relationship between POS and job burnout, as well as the role of regulatory emotional self-efficacy (RES) in moderating the above mediating process.

**Methods:**

A total of 784 police officers were surveyed with the POS Scale, the Job Burnout Questionnaire, the RES Scale, and the Minnesota Job Satisfaction Questionnaire.

**Results:**

(1) After controlling for gender, seniority, age, police classification, education, and marital status, regression analysis showed a significant negative correlation between POS and burnout (*r* = −0.42, *p* < 0.01), and the former had a significant negative predictive effect on job burnout (β = −0.42, *p* < 0.001). (2) The mediating effect test shows that job satisfaction plays a partial role in mediating the relationship between POS and job burnout. (3) Through the analysis of the moderated mediation model test, RES moderates the first half of the path of “POS → job satisfaction → job burnout.”

**Conclusion:**

POS not only directly affects police job burnout but also indirectly affects police job burnout through job satisfaction. RES enhances the influence of organizational support on job satisfaction. This study indicates the combined effect of POS, job satisfaction, and RES on job burnout and has certain guiding significance for alleviating police job burnout.

## Introduction

Job burnout is a comprehensive physical and psychological symptom caused by overwork stress, including emotional exhaustion, depersonalization, and decreased personal achievement (Maslach et al., 1996, 2001). It usually occurs in people who establish direct relationships with the target group with which they work and is the result of the interaction between individual personality and conditions in the environment (Maslach and Leiter, 2008). A large number of studies have shown that the job burnout rate of police officers was higher than that of other professionals, which is due to the unique organization, subculture, and work and life factors of police burnout (Schaible and Gecas, 2010; Schaible and Six, 2016). Job burnout can have many consequences for police officers: at the personal level, job burnout was related to police officers’ health, such as alcohol, drug abuse, and suicidal thoughts (Berg et al., 2003). At the family level, studies have confirmed that job burnout was associated with work-family conflict (Mikkelsen and Burke, 2004) and negative outcomes of police officers’ family members, such as anger and remoteness from family (Jackson and Maslach, 1982). At the level of social groups, job burnout affects the interaction between the police officers and the public and officers’ attitudes toward the use of violence (Kop et al., 1999), as well as their actual behavior in conflict situations (Euwema et al., 2004). In conclusion, studies on policing have consistently identified burnout as harmful to psychological health (Bhowmick and Mulla, 2016). Since job burnout has a negative impact on the individual, family, work, and society of police officers, especially in China, front-line police work is one of the most stressful and burdened occupations (Scoggins and O’Brien, 2015). Therefore, to better alleviate the job burnout of police officers and promote the harmonious coexistence of the police at work, in the family, and in society, it is necessary to study the job burnout of the Chinese police officers in depth.

## Theoretical Background

### The Influence of Organizational Support on the Job Burnout of the Police Officers

The core assumption of the Job Demands-Resources (JD-R) model (Bakker and Demerouti, 2017) is that each occupation has its own specific job characteristics associated with burnout. These job characteristics can be divided into two categories: job demands and job resources. Job demands refer to the areas of physical, emotional, or cognitive effort that need to be sustained at work (Demerouti et al., 2001). Job resources refer to physical, psychological, social, or organizational areas. They help employees achieve their work goals, reduce their job needs, and stimulate personal growth, learning, and development (Bakker and Demerouti, 2007). Another assumption of the JD-R model is that job characteristics lead to two different psychological processes: job demands initiate health damage processes and job resources initiate motivation processes. The effects of the two different psychological processes on the organization also differ. Research shows that job demands are the most important predictor of exhaustion and (lack of) job resources are the most important predictor of disengagement (Bakker et al., 2004). Job resources play a role in cushioning the impact of some job demands on job burnout (Bakker et al., 2005; Dubois et al., 2014).

Concerning work resources, perceived organizational support (POS) is an important psychological resource for job resources. POS is a subjective feeling that the organization cares about employees’ work dedication and welfare (Eisenberger et al., 1986). Organizational support theory (Rhoades and Eisenberger, 2002) argues that when employees feel that they have received good treatment from the organization in terms of management support, fairness and procedural justice, incentives, and working conditions, the sense of organizational support will increase.

Police job burnout is also deeply affected by departmental background and administrative policy (Adams and Mastracci, 2018). Related studies have proven that higher organizational support enhances organizational commitment and performance and reduces stress and turnover intention (Johnson, 2015; Cho and Song, 2017). Lower POS is a major predictor of burnout (Anomneze et al., 2016). Conservation of resource (COR) theory (Hobfoll, 2001) holds that access to additional resources can compensate for the loss of resources and help people reduce emotional exhaustion and enhance self-efficacy coping with job demands. POS can regulate the harmful effects of negative emotions and buffer work stress and dissatisfaction, because employees in collectivistic cultural contexts are more likely to consider POS as a meaningful coping resource (Hur et al., 2015). As an organizational resource, POS helps to relieve job stress and burnout (Olson et al., 2019).

In summary, high POS plays an important role in alleviating job burnout. However, how POS plays a role in job burnout and the conditions under which it takes effect are still unclear, and the moderated mediation model is helpful to solve this problem.

### The Mediating Effect of Job Satisfaction

Job satisfaction is a positive emotional state that comes from an individual’s subjective experience of his or her work (Judge and Bono, 2001). There is a significant positive correlation between POS and job satisfaction (Miao, 2011; Hashish, 2015; Chenga and Yang, 2018). There is evidence that organizational support improves police officers’ job satisfaction (Masal and Vogel, 2016; Vinod Kumar, 2017). POS can be seen as an organizational resource, and as an individual supplemental resource, POS can generate a range of positive emotional perceptions and experiences in the workplace (Riggle et al., 2009). These positive emotional experiences can replenish resources consumed by emotional labor and bring higher job satisfaction to employees (Wen et al., 2019).

On the other hand, job satisfaction also has a significant negative effect on job burnout, which is a predictive factor of job burnout (Kim et al., 2018; Wang et al., 2020). When employees feel the support of the organization, they continue to improve their job satisfaction. According to social exchange theory and the principle of reciprocity, employees with high POS feel obliged to respond to the organization with a positive working attitude and beneficial organizational behavior (Allen et al., 2003). They believe that their work is valuable, and they become more engaged in their work and willing to pursue higher goals, thereby reducing their willingness to leave and reduce job burnout. In contrast, when the company policy, management measures, wages and benefits, material working conditions, status, and other factors are missing, individuals will be dissatisfied with their work (Herzberg et al., 1959). People who are not satisfied with their jobs tend to have higher burnout scores (Cagan and Gunay, 2015; Kim et al., 2019). Such individuals feel less organizational care and job satisfaction, which lead to loss of enthusiasm for work and burnout.

Thus, the current study suggests that POS affects an individual’s psychological state (job burnout) through emotional experience (job satisfaction); that is, job satisfaction plays a mediating role in POS and job burnout. Therefore, this study proposes:

*Hypothesis 1*: *job satisfaction plays a mediating role in perceived organizational support and job burnout.*

### The Moderating Effect of Regulatory Emotional Self-Efficacy

Studies have shown that POS can significantly predict job burnout, and high POS can reduce job burnout (Olson et al., 2019). Some studies have also found that although there was a correlation between the two, the relationship was not significant (Xu et al., 2017). The differences in the results of previous studies suggest that the direct/indirect relationship between POS and job burnout may be conditional or moderated by some factors. In the case of this study, the test of the mediating effect of job satisfaction can explain the mechanism of POS on the job burnout, but the mediating effect would be different in different situations, that is, under the influences of other factors. Studies have shown that job satisfaction is influenced by both external factors such as working conditions, colleagues, and types of support gained, and internal factors, such as self-efficacy beliefs (Lee and Quek, 2017; Troesch and Bauer, 2017).

Traditionally, scholars have divided the influencing factors of burnout into two categories: situational factors and individual factors (Bakker et al., 2014). Personal resources are positive self-assessments associated with resilience, referring to the individual’s ability to successfully control and influence the environment (Hobfoll et al., 2003). This positive self-evaluation predicts goal setting, motivation, performance, job and life satisfaction, and other desirable outcomes (Judge et al., 2004). Brackett (2010) found that emotional regulation ability can predict less exhaustion and more positive emotions, and improve personal achievement and job satisfaction. Studies have also shown that manipulation of the belief that one is better able to manage emotional states leads to less negative affective reactivity (Benfer et al., 2018). Workers who do not believe that they can control the emotions associated with recurrent daily hassles or serious struggles are unlikely to adapt to novel and unfamiliar situations, respond flexibly to stressful circumstances, or encounter life with curiosity and enthusiasm (Consiglio et al., 2013). When people face a challenging and high-pressure environment and cannot fully regulate their strong negative emotions, they improperly externalize the negative emotions (Eisenberger et al., 2001). Experiencing positive emotions can enhance cognitive function, buffer the interference effect of aversion experience, and promote adaptive coping (Folkman and Moskowitz, 2000).Therefore, people’s regulatory emotional self-efficacy (RES) contributes to personal mental health and emotional comfort (Caprara et al., 2008). The present study will explore the regulatory effect of RES on the mediation path of POS → job satisfaction → job burnout.

**FIGURE 1:**
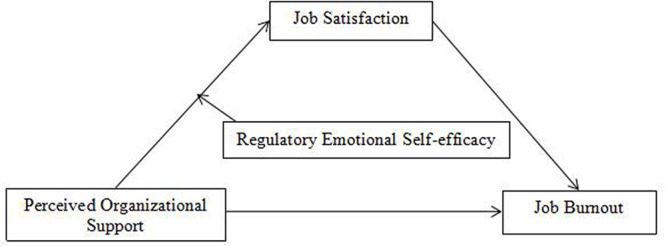
Hypothesized model.

Bandura et al. (2003) believe that RES is the degree of confidence in the ability to effectively regulate one’s own emotional state and manage one’s own emotions. RES includes the efficacy of expressing positive emotions and the efficacy of regulating the negative emotions of pain and anger. According to Fredrickson’s (2004) broaden-and-build theory, positive emotions broaden one’s thought-action repertoire and build resources. The experience of positive emotions increases the individual’s likelihood of finding positive meaning in subsequent events (Fredrickson, 2000); as a result, RES increases individual job satisfaction in work. However, negative emotions narrow one’s thought-action repertoire and deplete resources (Fredrickson et al., 2008). Studies have shown that RES can positively predict individual job satisfaction, and employees with high RES can effectively express positive emotions and manage negative emotions and increase job satisfaction. Heuven et al. (2006) believe that high RES can weaken the negative effects of negative emotions and ensure employees’ enthusiasm for work, while low RES can lead to emotional exhaustion and a decrease in work enthusiasm. Therefore, this study proposes:

*Hypothesis 2*: the mediating effect of job satisfaction on the relationship between POS and job burnout is moderated by RES. In summary, this study constructs a moderated mediation model, as shown in Figure 1.

## Materials and Methods

### Participants

A convenience sampling method was adopted to send out questionnaires to 850 police officers from different provinces of China. A total of 784 valid questionnaires were collected, and the effective rate was 92.24%. There were 587 men, 173 women, and 24 respondents that did not report gender. The proportion of policewomen in this study is 28.83%, which is higher than the national average according to the proportion of about 10% of policewomen recruited by Chinese public security organization every year (Ma, 2019). The average length of service was 10.89 years (*SD* = 9.09) varying from 1 to 42 years and the average age was 32.86 years (*SD* = 10.014) varying from 17 to 60 years.

### Measures

#### Perceived Organizational Support Scale

The Survey of Perceived Organizational Support (POS) was developed by Eisenberger et al. (1986). The simplified version consists of the nine items with the highest load in the original scale (of which 4 and 6 are reverse questions). Items include “The organization is very little concerned about me” or “management really care about my happiness.” The scale was scored at seven points (1 = *totally disagree*, 7 = *totally agree*). The higher the score was, the higher the POS. The results of confirmatory factor analysis showed that the construct validity was good: χ*^2^/df* = 2.96, *CFI* = 0.90, *NNFI* = 0.90, *RMSEA* = 0.076 (Ling et al., 2006). In this study, the Cronbach’s α coefficient of the scale was 0.75.

#### Job Burnout Questionnaire

The Chinese version was revised by Li and Shi (2003) according to Maslach and Schaufei’s MBI-GS. The scale consists of three subscales of emotional exhaustion (four items, e.g., “Work makes me feel physically and mentally tired”), cynicism (four items, e.g., “I am becoming less and less concerned about whether I have contributed to the work I have done”), and reduced personal achievement (six items, e.g., “I am confident that I can finish all the work effectively”). All items were rated on a seven-point Likert scale (0 = *never*, 6 = *always*). The score of the subscale was the sum of all the items in this dimension. All the items with low achievement were scored in reverse. Higher scores of emotional exhaustion and cynicism, and lower scores of reduced personal achievement, indicated stronger job burnout. The results of confirmatory factor analysis showed that the construct validity was good: χ*^2^/df* = 3.99, *CFI* = 0.92, *GFI* = 0.91, *RMSEA* = 0.08 (Li and Shi, 2003). In this study, the Cronbach’s α coefficient of the scale was 0.83.

#### Regulatory Emotional Self-Efficacy Scale

The Chinese version of RES revised by Caprara et al. (2008) was used. This scale included items on perceived capability to express positive affect (one to four items, e.g., “When something happens, I express my pleasure”) and to regulate negative affect (five to nine items, e.g., “In the face of difficulties, I am able to be discouraged.”) and angry affect (10–12 items, e.g., “I can avoid annoyance when others deliberately bother me”). The scale was composed of 12 items, and the five-point scoring method (1 = *total non-conformity*, 5 = *total conformity*) was adopted. The higher the score was, the better the individual’s RES. The results of confirmatory factor analysis showed that the construct validity was good: χ*^2^/df* = 4.92, *CFI* = 0.91, *GFI* = 0.95, *RMSEA* = 0.069 (Wen et al., 2009). In this study, the Cronbach’s α coefficient of the scale was 0.894, and the Cronbach’s α coefficients of each subscale were 0.86, 0.83, 0.82.

#### Minnesota Satisfaction Questionnaire

The 20-item Minnesota Satisfaction Questionnaire was developed by Weiss et al. (1967). A five-point Likert scale (1 = *very dissatisfied*, 5 = *very satisfied*) was used to rate the items (“Are you satisfied with the busyness of your current work?”), with higher scores indicating higher job satisfaction. The results of confirmatory factor analysis showed that the construct validity was good: *CFI* = 0.97, *AGFI* = 0.94, *RMSEA* = 0.06, *SRMR* = 0.03 (Lu, 2019). In this study, the Cronbach’s α coefficient of the scale was 0.93.

### Research Procedures and Data Processing

During the period of police mass learning, graduate students majoring in psychology were taken as the subjects, and a questionnaire was used to carry out the collective test. The subjects were first introduced the purpose of the study, and then asked to sign informed consent (which stated that the survey was conducted anonymously and that the results were for academic research only, and that all information would be kept confidential), read the instructions, and complete the questionnaire independently according to their true situation. All the participants completed the questionnaire within 25 min, and the questionnaires were collected immediately after completion. This study was approved by the Ethics Committee of the School of Psychology of Jiangxi Normal University.

The collected data were sorted and analyzed by SPSS 23.0 and its macro program PROCESS 2.13 (Hayes, 2013). The SPSS macro program can verify a variety of mediated moderation and moderated mediation models based on the bootstrap method of deviation correction percentile. By taking 5000 bootstrap samples (each sample size was 784 people), the robust standard error and bootstrap confidence interval of parameter estimation were obtained. If the confidence interval did not contain 0, the result was considered significant (Erceg-Hurn and Mirosevich, 2008).

## Results

### Common Method Biases

The data collected in this study were all self-reported by the participants, and there may be common methodological biases. To reduce the influence of biases on the results of the study, in terms of program control, this study adopted anonymous investigation, and some items were scored in reverse. Statistically, the Harman single factor model method was used to test the common method biases of the data (Podsakoff et al., 2012). The results show that there were 10 factors with eigenvalues greater than 1, and the variation explained by the first factor was only 27.539%, less than the critical value of 40%, which indicates that there was no serious common method bias problem in each variable in this study.

### Descriptive Statistics and Correlation Analysis

Descriptive statistics and correlation analysis were carried out on POS, job burnout, job satisfaction, and RES. POS was positively related to job satisfaction and RES. POS, job satisfaction, and RES were negatively related to job burnout. Job satisfaction was positively related to RES (see Table 1).

**TABLE 1:** Means, standard deviations, and correlations among study variables.

	*M*	*SD*	1	2	3
1 Perceived organizational support	45.52	10.685	–		
2 Job burnout	41.80	6.502	−0.417**	–	
3 Job satisfaction	72.35	11.803	0.637**	−0.356**	–
4 Regulatory emotional self-efficacy	44.53	7.604	0.465**	−0.412**	0.581**

N = 784; **P < 0.01. The following are the same.

### The Difference Test of Demographic Variables

Independent sample *T*-test was used to test the gender difference, and single factor variance analysis was used to analyze the variance of age, length of service, police classification, marital status, and educational level (see Table 2): (1) There was a significant difference in job satisfaction between men and women (*t* = 5.01, *p* < 0.001), and the average score of job satisfaction of women was higher than that of men, which indicated that the job satisfaction of women was significantly higher than that of men. (2) POS, job satisfaction, and RES all differed significantly by police classification (*F* = 12.89, *p* < 0.001; *F* = 3.50, *p* < 0.01; *F* = 13.87, *p* < 0.001; *F* = 3.22, *p* < 0.01). From the point of view of the average score of POS and RES, the criminal police scored the highest; from the average score of job burnout, the criminal police scored the lowest; from the point of view of job satisfaction, the traffic police scored higher. (3) The Main variables differed by marital status (*F* = 13.35, *p* < 0.001; *F* = 3.52, *p* < 0.01; *F* = 15.44, *p* < 0.001; *F* = 3.80, *p* < 0.01). In terms of POS, job satisfaction, and RES, the scores of unmarried respondents were higher than the scores of married respondents, but average score of job burnout was lower for unmarried respondents. (4) POS, job satisfaction, and RES were all significantly different by educational level (*F* = 8.57, *p* < 0.001; *F* = 3.35, *p* < 0.01; *F* = 4.93, *p* < 0.001). Except for RES, respondents with a graduate degree scored lower than the respondents with below high school education. With the increase in educational level, the scores of POS and job satisfaction also increased. (5) The main variables have significant differences in age and length of service. On the whole, with the increase in age and length of service, the scores of POS, job satisfaction, and RES decreased, and the scores of job burnout increased. Therefore, in the follow-up mediated regulation analysis, gender, age, length of service, police classification, marriage status, and educational level are controlled.

**TABLE 2:** The difference test of the demographic variables of each variable.

		Perceived organizational						Regulatory emotional	
		support		Job burnout		Job satisfaction		self-efficacy	
		*M* ± *SD*	*t/F*	*M* ± *SD*	*t/F*	*M* ± *SD*	*t/F*	*M* ± *SD*	*t/F*
Gender	Male	45.30 ± 10.71	0.20	41.91 ± 6.39	3.68	71.57 ± 11.98	5.01***	44.20 ± 7.59	0.14
	Female	46.40 ± 10.38		41.82 ± 6.78		74.98 ± 10.70		45.40 ± 7.32	
Police classification	Criminal police	48.55 ± 10.08	12.89***	40.19 ± 6.01	3.50**	74.72 ± 12.81	13.87***	45.96 ± 7.04	3.22**
	Traffic police	48.40 ± 8.94		42.28 ± 6.31		76.42 ± 9.25		44.76 ± 7.40	
	Registrar police	46.15 ± 10.82		41.89 ± 7.96		73.92 ± 12.80		44.79 ± 8.46	
	Patrol	46.89 ± 9.00		41.81 ± 6.48		72.19 ± 9.43		44.04 ± 7.35	
	Firefighter	40.56 ± 5.43		45.33 ± 4.03		72.89 ± 10.89		39.11 ± 8.25	
	Others	42.04 ± 11.05		42.53 ± 6.23		68.25 ± 11.06		43.54 ± 7.45	
Marital status	Married	43.67 ± 11.29	13.35***	41.77 ± 6.30	3.52**	69.89 ± 12.06	15.44***	44.00 ± 7.66	3.80**
	Unmarried	48.99 ± 8.69		41.59 ± 6.23		76.54 ± 10.35		45.61 ± 7.29	
	Divorced	44.61 ± 9.70		42.12 ± 8.72		68.00 ± 11.93		43.39 ± 9.38	
	Widowed	40.11 ± 10.55		48.11 ± 8.87		72.33 ± 8.11		41.67 ± 5.83	
	Remarriage	39.79 ± 8.13		45.64 ± 8.47		72.50 ± 6.49		39.79 ± 6.27	
Education level	High school below	28.36 ± 9.92	8.57***	43.10 ± 6.35	0.22	63.10 ± 13.48	3.35**	34.91 ± 7.19	4.93***
	College	43.55 ± 11.14		42.17 ± 6.99		69.77 ± 11.60		43.41 ± 7.47	
	Undergraduate	46.10 ± 10.36		41.69 ± 6.30		72.99 ± 11.78		44.96 ± 7.47	
	Postgraduate	48.92 ± 8.42		41.73 ± 6.20		73.18 ± 11.09		43.84 ± 7.91	
Age	17–60		4.36***		2.21***		4.13***		2.39***
Length of work	1–42		3.97***		1.39		1.86***		4.36***

**P < 0.01, ***P < 0.001.

### The Mediation Effect of Job Satisfaction

**FIGURE 2:**
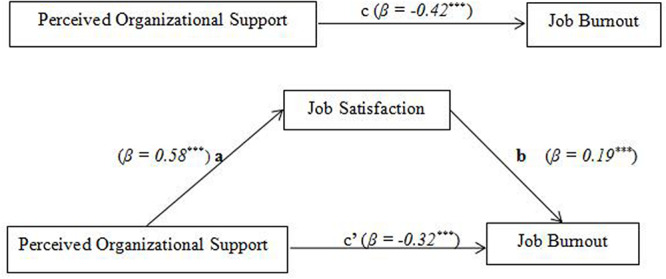
The mediating role of job satisfaction between perceived organizational support and job burnout. ***P < 0.001.

Since the mediated effect estimates are not normally distributed, the study uses the non-parametric percentile bootstrap method of the bias correction to perform the mediation test. First, after controlling for gender, age, length of service, police classification, marital status, and educational level, a regression analysis of path C organizational support for job burnout was performed (see Figure 2). The results showed that the contribution of organizational support to job burnout was 17.4%, *F* = 164.004, and the regression equation was significant. The standardized regression equation coefficient Beta of organizational support was −0.417, which indicated that organizational support could negatively predict job burnout (see Table 3).

**TABLE 3:** Regression equation of organizational support to job burnout.

	Model	β	*T*	*R*^2^	adjusted *R*^2^	*F*
1	Perceived organization support	−0.42	−12.81***	0.174	0.173	164.004***

***P < 0.001.

Then, all variables were normalized, and model 4 of the SPSS macro program PROCESS (version 2.13) was used to verify the mediating effect of job satisfaction. Testing paths a, b, and c’ (Figure 2), the results show that the path coefficients of paths a (95% CI: 0.52, 0.64) and b (95% CI: −0.28, −0.10) are significant in the indirect effect, indicating that job satisfaction plays a significant mediating role between POS and job burnout, and the mediating effect model is established (see Table 4). At this time, the path coefficient c’ (95% CI: −0.42, −0.24) is also significant, indicating that POS can still significantly negatively predict job burnout. That is, job satisfaction partially mediates the relationship between POS and job burnout, and Hypothesis 2 is verified.

**TABLE 4:** Mediating effect of job satisfaction.

Path	Independent variable	Dependent variable	β	*t*	*p*	Lower CI	Upper CI
a	Perceived organizational support	Job satisfaction	0.58	18.40	0.0000	0.52	0.64
b	Job satisfaction	Job burnout	–0.19	–4.06	0.0001	–0.28	–0.10
c’	Perceived organizational support	Job burnout	–0.323	–7.02	0.0000	–0.42	–0.23
Indirect	a → b → c’		–0.11			–0.17	–0.05

### The Moderating Role of Regulatory Emotional Self-Efficacy

**FIGURE 3:**
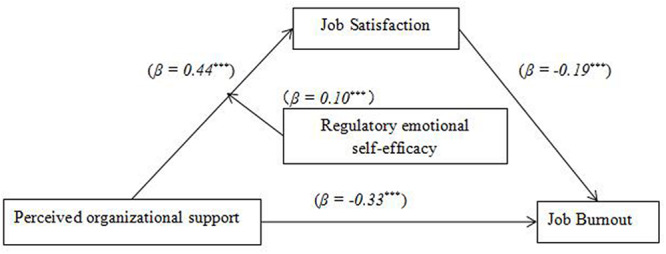
Moderated mediated model. ***P < 0.001.

Model 7 of the SPSS Macro Program PROCESS (version 2.13) was used to test the moderating effect of RES. According to the method proposed by Wen and Ye (2014), the relationship between POS and job burnout, the mediating role of job satisfaction, and RES are discussed (see Table 5). With job satisfaction as the dependent variable, POS significantly positively predicted job satisfaction (β = 0.44, *p* < 0.001), RES significantly predicted job satisfaction (β = 0.34, *p* < 0.001), and the interaction between POS and RES significantly positively predicted job satisfaction (β = 0.10, *p* < 0.001). This shows that RES moderates the first half of the mediating role between POS and job burnout. In the second equation, with job burnout as the dependent variable, job satisfaction significantly negatively predicted job burnout (β = −0.19, *p* < 0.001), POS significantly negatively predicted job burnout (β = −0.30, *p* < 0.001). According to the results of moderated mediated effects, RES regulates the first half path of the mediation process. Therefore, RES plays a moderating role in the first half of the mediating effect, and Hypothesis 2 is verified. The specific model is shown in Figure 3.

**TABLE 5:** Regression analysis results of regulatory emotional self-efficacy moderate the mediation process.

Regression equation		Overall model fit			Significance of regression coefficient			
Outcome	Predictor	*R*	*R*^2^	*F*	β	*t*	*LLCI*	*ULCI*
JS	Age	0.74	0.54	85.97***	0.01	1.04	–0.01	0.03
	Gender				0.10	1.61	–0.02	0.23
	Length of service				–0.02	–2.23	−0.04**	–0.01
	Marital status				0.13	3.76	0.06***	0.20
	Classification				–0.03	–2.33	−0.06**	–0.01
	Education level				0.01	0.17	–0.08	0.09
	POS				0.44	13.97***	0.38	0.51
	RES				0.34	11.24***	0.28	0.40
	POS × RES				0.10	3.97***	0.05	0.15
JB	Age	0.47	0.22	22.65***	0.02	1.30	–0.01	0.04
	Gender				0.01	0.03	–0.16	0.17
	Length of service				–0.02	–1.77	–0.05	0.01
	Marital status				0.18	3.89***	0.09	0.27
	Classification				0.01	0.47	–0.03	0.04
	Education level				0.07	1.24	–0.04	0.18
	POS				–0.37	−7.06***	–0.42	–0.24
	JS				–0.19	−4.05***	–0.28	–0.10

POS = perceived organizational support; JS = job satisfaction; RESE = regulatory emotional self-efficacy; JB = job burnout; LLCI = lower level confidence interval; ULCI = upper level confidence interval. **P < 0.01, ***P < 0.001.

To reveal more clearly how RES can regulate the influence of POS on job satisfaction, by adding or subtracting a standard deviation, RES is divided into high and low groups. The mediating effect of job satisfaction between POS and job burnout and its 95% bootstrap confidence interval were calculated as shown in Table 6.

**TABLE 6:** The mediating effect of regulatory emotional self-efficacy between the perceived organizational support and the job burnout.

Regulatory emotional self-efficacy	Conditional indirect effect	*SE*	Lower CI	Upper CI
*M - SD*	0.33	0.04	0.26	0.41
*M*	0.42	0.03	0.36	0.49
*M* + *SD*	0.52	0.04	0.43	0.60
				

**FIGURE 4:**
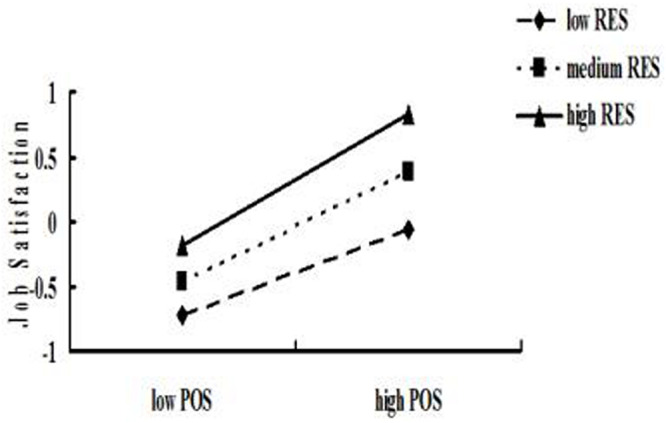
Simple slopes of regulatory emotional self-efficacy moderate the relationship perceived organizational support between and job satisfaction.

Furthermore, the simple slope test was used to analyze the moderating effect. According to RES, an interaction diagram was drawn (see Figure 4). With the increase in RES, the positive predictive effect of POS on job satisfaction increased (from β = 0.33, *p* < 0.001 to β = 0.52, *p* < 0.001). Therefore, RES can promote the positive predictive effect of POS on job satisfaction.

## Discussion

### The Relationship Between Police Perceived Organizational Support and Job Burnout

The present study found that the overall level of job burnout of Chinese police officers is low to moderate, which may be related to the diversity of the subjects in this study and the different job content and responsibilities of police officers in different positions. The police samples with moderately severe job burnout found in the study are prison guards. These samples may present moderately severe job burnout because the work content of prison guards is unvaried, the environment is monotonous, the pressure is great, and most of the people they contact are criminals with personality abnormalities and psychological abnormalities, which can easily lead to a low sense of achievement and exhaustion. The generally low rate of burnout may be because that the average age of the subjects in this study is fairly young. Young people in their early 30s tend to have better physical fitness than old people. Many studies of physical cognition show that people with strong body muscles tend to have stronger psychological quality and better ability to withstand pressure.

The results of the correlation analysis show that there is a significant negative correlation between POS and job burnout. The regression analysis showed that POS had a significant negative predictive effect on job burnout after controlling for age, gender, length of service, police type, marriage status, and educational level. The job burnout of individuals with high POS is lower. This is consistent with the findings of previous studies (Altinoz et al., 2016; Miller et al., 2017) that POS has a negative predictive effect on job burnout. Based on the JD-R model, job resources trigger motivation processes, and when employees have sufficient available resources at work, they produce an incentive response to their work, manifested as energy, dedication, and concentration (i.e., work engagement; Schaufeli and Bakker, 2004). Employees are able to complete their jobs in a positive condition, and job burnout is reduced accordingly (Yamazaki et al., 2000). POS is a valuable resource that can enhance employees’ confidence in responding to their role needs (Lazarus, 1991). POS focuses on the support provided by the organization to help employees perform effectively and handle stressful situations adequately (Stinglhamber and Vandenberghe, 2003) and such organizational support meets their socioemotional needs (Duke et al., 2009). In the Chinese police culture of “asking the police for help,” police officers are given a high sense of mission, and they spend long periods in a state of “high pressure, high risk and high stress.” As predicted by COR theory, the organization provides employees with assistance so that they can maintain good working conditions, thereby reducing the physical and mental consumption of resources and burnout at work, enabling them to strive and focus on their tasks (Lan et al., 2020).

### The Mediating Role of Job Satisfaction

The results of this study show that job satisfaction plays a partial mediating role between POS and job burnout. High POS is helpful for improving police job satisfaction. Job satisfaction can have a significantly negative impact on job burnout. Therefore, it can alleviate the job burnout of police officers.

The results verified the positive effect of POS: there was a significant negative relationship between POS and police job burnout, which is consistent with previous findings (Yaghoubi et al., 2014; Kula, 2016), and this negative relationship can be explained by job satisfaction. According to the JD-R model, employees’ work behavior can improve employees’ job satisfaction and reduce job burnout by promoting the achievement personal goals at work (that is, increasing personal resources, such as self-efficacy) and actively creating a richer work environment (that is, increasing work resources, such as organizational support). POS, as a resource in the organization, can enable employees to generate a series of positive emotions based on the support and understanding of the organization and the affirmation of their abilities. This positive emotion helps restore the emotional exhaustion of employees in emotional labor (Wen et al., 2019).

The results of this study are consistent with the theory of organizational support (Eisenberger et al., 1986). The care and respect given by the organization to employees is very important, and POS is the key to hard work. As for the police, when they are well treated by the organization, their willingness to devote efforts to their work comes from their inner awareness and their noble spirit of dedication (Liu et al., 2019). This not only enhances the performance of the organization but also yields a corresponding reward in the principle of reciprocity, so their job satisfaction is also enhanced (Rhoades and Eisenberger, 2002). On the other hand, the improvement of job satisfaction can alleviate job burnout. A high level of POS improves job satisfaction and relieves job stress and burnout (Appelbaum et al., 2019); conversely decreasing work satisfaction can cause a decrease in work productivity and an increase in burnout (Yilmaz, 2018). From the COR theory, it can be seen that job burnout occurs when an individual cannot replenish resources after investing many resources (and sometimes suffering a slight, long-term loss), needs to invest time and energy, and borrows from family time and intimacy to support the job (Hobfoll, 2001). The high and low work requirements and the gain and loss of working resources are the key factors that affect job burnout, and the imbalance of work requirements and working resources greatly influence an individual’s enthusiasm and physical and mental health (Hobfoll, 1988, 1989). The job satisfaction of individuals experiencing an imbalance between job requirements and work resources is lower than that of individuals with a balance; this is because when individuals are faced with the dual pressure of low POS and high job requirements, they need to mobilize their existing resources and self-control to deal with it. In this process, their own resources are constantly consumed, which leads to the reduction of job satisfaction. Individuals need constant self-intervention to cope with reduced job satisfaction. If individuals must frequently use organizational resources or self- intervention to improve their work, the continuous monitoring and efforts to change emotions exhaust their psychological resources and lead to the buildup of negative emotions internally, leading to draining resources and job burnout (Grandey and Gabriel, 2015).

### The Moderating Effect of Regulatory Emotional Self-Efficacy

This study found that RES can promote the positive predictive effect of POS on police job satisfaction. Based on the explanation of the JD-R model, POS, a working resource, affects job satisfaction through RES. That is, at the same level of POS, the job satisfaction of the police officers with high RES was higher than that of police officers with low RES. The reason for the positive influence of experience is that individuals with a high level of RES are more confident that they can effectively express positive emotions and manage negative emotions. Even when faced with frustration and adverse conditions, they adjust themselves quickly, thus reducing the possibility of self-injury, reducing negative emotional experience, and in turn enhancing happiness or job satisfaction (Caprara and Steca, 2005; Jin et al., 2020; Liu et al., 2020). However, if individuals do not believe that they can control their emotions, it is more difficult for them to truely control their emotions; ultimately, this will affect their mental health (Azizli et al., 2015).

The establishment of supportive relationships can enhance personal efficacy, which in turn affects the quality of emotional and behavioral functions. Social support can produce beneficial results only by improving the degree of coping self-efficacy. Moreover, the expression of positive and negative emotions generally has different social effects (Bandura, 2002). In this study, the police officers who were supported by the organization and had high RES could adjust themselves more quickly, and experience less negative emotions and more positive emotions. Positive emotion can expand people’s thought-action reserve, and broadened mindsets bring individuals indirect and long-term adaptive benefits by enabling them to build enduring personal resources (Fredrickson, 2004), better finish the job and improve job satisfaction. Individuals with more resources are less vulnerable to resource loss (Hobfoll et al., 2018) and police with low emotional self-efficacy are more likely to vent their negative emotions, which affects personal development and achievement (Bandura et al., 2003) and thus reduce job satisfaction. This is consistent with the JD-R model. RES as a personal resource is also an important predictor of motivation, which can mitigate the adverse effects of job demands (thus reducing job satisfaction).

## Limitations and Future Research Suggestions

This study revealed that POS affects police job burnout via a moderated mediation. This verified that multiple factors affect job burnout. Therefore, to achieve the best intervention effect and minimize police job burnout, future interventions cannot focus only on one aspect but must be integrated and systematic intervention, starting from three aspects (POS, job satisfaction, and RES).

There are limitations to this study. First, as a cross-sectional study, this study was unable to investigate the causal relationship between variables, and future scholars could make longitudinal studies, and seek to determine a causal relationship between the variables. Second, the data of the study come from self-reported information. Future research should integrate other information channels to collect data, such as the parents, colleagues of respondents. Different sources of information can confirm the data each provides, and more objective measurements can be obtained. Third, no data coming from the family and the broader police working environments which are also affected by police job burnout have been taken into consideration, such as family work conflict. Last, this study only investigated the Chinese police. The results could be different for police officers of different cultural backgrounds, so whether the results of this study can be generalized to the police officers with non-Chinese cultural backgrounds remains to be tested.

## Conclusion

Based on our analysis and discussion, the current study suggests that POS affects directly the police job burnout as well as indirectly through job satisfaction. After controlling for gender, age, length of service, police classification, marital status, and educational level, job satisfaction plays a partial mediating role between POS and job burnout. According to the results of this study, the role of job satisfaction in POS and job burnout is moderated by RES. For police officers with high RES, the promoting effect of POS on job satisfaction is enhanced.

## Data Availability Statement

The data are available on request.

## Ethics Statement

This study was approved by the Ethics Committee of the School of Psychology of the Jiangxi Normal University. The patients/participants provided their written informed consent to participate in this study.

## Author Contributions

XQZ and JL contributed to conception and design of the study. XXZ performed the statistical analysis and wrote the first draft of the manuscript. XQZ and MC revised it critically for important intellectual content. CW collected the raw data and organized the database.

## Conflict of Interest

The authors declare that the research was conducted in the absence of any commercial or financial relationships that could be construed as a potential conflict of interest.
